# Comparison of femtosecond laser-assisted deep anterior lamellar keratoplasty and penetrating keratoplasty for keratoconus

**DOI:** 10.1186/s12886-015-0140-x

**Published:** 2015-10-27

**Authors:** Yueqin Chen, Dan-Ning Hu, Yuan Xia, Liping Yang, Chunyan Xue, Zhenping Huang

**Affiliations:** Department of Ophthalmology, Jinling Hospital, School of Medicine, Nanjing University, Nanjing, 210002 China; Tissue Culture Center, Departments of Pathology and Ophthalmology, The New York Eye and Ear Infirmary, New York Medical College, New York, NY 10003 USA

**Keywords:** Femtosecond laser-assisted deep anterior keratoplasty, Femtosecond laser-assisted penetrating keratoplasty, Keratoconus, Baring Descemet’s membrane

## Abstract

**Background:**

To compare outcomes of femtosecond laser-assisted deep anterior lamellar keratoplasty (FSL-DALK) and penetrating keratoplasty (FSL-PK) for the treatment of keratoconus.

**Methods:**

Twenty eight eyes underwent FSL-DALK (consisted of 12 eyes in the FSL-DALKa subgroup without baring the Descemet’s membrane and 16 eyes in the FSL-DALKb subgroup baring the Descemet’s membrane using big-bubble technique) were compared with 12 eyes that underwent FSL-PK for keratoconus. These patients underwent an ophthalmic examination preoperatively and 3, 6, 9, and 12 months postoperatively.

**Results:**

The postoperative BCVA in the FSL-PK group, and the FSL-DALKb subgroup were significantly better than that in the FSL-DALKa subgroup (*P* < 0.05), whereas no differences were found between the FSL-DALKb subgroup and the FSL-PK group (*P* > 0.05). There were no significant differences in the mean spherical equivalent (SE) and astigmatism between the FSL-DALK and the FSL-PK groups, nor between the subgroups of FSL-DALK during the follow-up period (*P* > 0.05). At the last follow-up, the mean endothelial cell loss in the FSL-DALK group (9.12 %) was significantly less than that in the FSL-PK group (20.79 %) (*P* < 0.001), while there was no difference between the FSL-DALKa (9.15 %) and the FSL-DALKb (9.10 %) subgroups (*P* = 0.15). The FSL-DALK group seemed to have fewer graft rejections (1/28 cases) than the FSL-PK group (2/12 cases), although Kaplan-Meier curve showed no significant difference between the two groups (*P* = 0.144).

**Conclusions:**

In this retrospective study, the results suggested that FSL-DALKb gives better visual outcome, and FSL-DALKb is a better option for keratoconus whose endothelium is not compromised. However, larger and prospective studies are further required.

## Background

Penetrating keratoplasty (PK) has been considered as the definitive procedure for the treatment of keratoconus over the past few decades [1, 2]. However, it can be complicated by allograft endothelial rejection with subsequent risk of graft failure [3]. Deep anterior lamellar keratoplasty (DALK), which involves the removal of anterior diseased cornea while leaving deeper tissue intact, offers the advantages of reducing the risks of graft rejection and intraocular complications. However, it is technically more challenging and may result in the loss of best-corrected visual acuity (BCVA) due to the irregular stromal interface between the donor and the recipient [4]. Many improvements have evolved to minimize these difficulties, such as automated microkeratomes and big-bubble technique [5–7].

Femtosecond laser technology, that allows corneal tissue to be cut without thermal or shockwave damage to the surrounding tissue, has been recently introduced in the corneal surgery. The technology facilitates the preparation of donor and recipient tissue for the anterior, posterior, and penetrating keratoplasty [8]. It is able to achieve greater precision and accuracy than conventional and manual trephination techniques, and thus lead to less interface irregularity and haze. These features may result in faster and better visual rehabilitation [9, 10].

In this study, we aimed to compare outcomes of femtosecond laser-assisted PK (FSL-PK) and two subgroups of femtosecond laser-assisted DALK (FSL-DALK) in keratoconus, that is, with and without baring Descemet’s membrane (DM). To our knowledge, this is the first study that compared FSL-PK with two different FSL-DALK surgical approaches distinguishing total removal of recipient bed stroma from retention of recipient stroma, thus enabling a clearer view of the evolution of FSL-DALK techniques and the difference in their outcomes in comparison with FSL-PK.

## Methods

### Design

A total of 44 keratoconus eyes of 40 patients underwent femtosecond laser-assisted (FSL) keratoplasty between February of 2012 and October of 2013 at the Refractive Center, Department of Ophthalmology, Jinling Hospital, Nanjing, China. Of the 44 eyes, 4 eyes that had incomplete follow-up were excluded. The remaining 40 cases were evaluated in this retrospective, comparative study. Of these 40 eyes, 28 underwent FSL-DALK (consisted 12 eyes in the FSL-DALKa subgroup without baring the Descemet’s membrane and 16 eyes in the FSL-DALKb subgroup baring the Descemet’s membrane using big-bubble technique) and 12 underwent FSL-PK.

The diagnosis of keratoconus was made clinically from the history, slit-lamp examination, keratometry, and refraction. The indication for keratoplasty was poor functional vision and intolerance of other methods for optical correction such as spectacles and rigid gas permeable contact lenses.

The study protocol was approved by the Ethical Committee of Jinling Hospital. All patients were fully informed of the details and possible risks of the procedure, and written informed consents were obtained from all the participated patients. Described research adhered to the tenets of the Declaration of Helsinki.

### Ophthalmic examination

Preoperative and postoperative evaluations included slit-lamp examination, indirect fundus examination, best-corrected visual acuity (BCVA), intraocular pressure, manifest refraction, corneal topography (CAS; EyeSys, Houston, TX, USA), endothelial cell density (ECD) (SP-3000P, Topcon Corp.), and anterior segment ocular coherence tomography (OCT; Visante OCT, Carl Zeiss Meditec, Inc., Dublin, CA). Postoperative examinations were performed at 3, 6, 9, and 12 months after the surgery.

### Surgical technique

The surgeries were all performed by the same surgeon (Zhenping Huang). Patients received 4-ml of 2 % xylocaine and 0.5 % bupivacaine as a combination of retrobulbar anesthesia. The 500-kHz VisuMax femtosecond laser system (Carl Zeiss Meditec, Inc., Dublin, CA) was used to trephine both recipient and donor corneas. After that, the patient was wheeled from the femtosecond laser into the operating room and the eye was prepared and draped in the usual sterile fashion for intraocular surgery. To create the donor graft, corneoscleral donor tissue was mounted on a Barron artificial anterior chamber. When an entire donor globe was available, it was mounted directly under the laser. Laser settings used were as follows: trephination diameter 7.3 to 8.1 mm with donor oversized by 0.2 mm, thickness 300 to 510 μm with up to 20 % additional thickness added depending on the edema of the donor, spiral method, 230 to 240 nanojoules spiral energy, 230 to 240 nanojoules side cut energy, side cut angle 90°, spot separation 1.9 μm, layer separation 1.9 μm.

### FSL-DALKa Group

The FSL incision depth of the recipient was programmed to begin 70 μm anterior to the endothelium at the thinnest point on the optical coherence tomography pachymetry map (Visante, Carl Zeiss Meditec, Inc., Dublin, CA). The donor was trephined using similar settings except 0.2 mm oversized diameter. After trephination, the edge of the recipient and donor corneal buttons was separated from the stromal bed with a Sinskey hook (Rhein Medical, Tampa, FL) and the posterior border of the button gently separated with the Siebel spatula (Rhein Medical, Heidelberg, Germany). The donor button was then placed on the recipient residual corneal stromal bed and sutured with 16 interrupted 10–0 nylon sutures.

### FSL-DALKb Group

The FSL trephination was formed to a depth of 50 to 60 % thickness in the recipient cornea. A 27-gauge Fogla needle attached to a 3-mL syringe with air (Storz Ophthalmics; Bausch & Lomb Incorporated, Vaughn, Ontario, Canada) was inserted bevel down into the paracentral cornea, parallel to the corneal surface, and advanced 2 to 3 mm along the corneal plane. An air-bubble was injected to separate the DM from the stroma. Scissors were used to remove the remaining overlying stroma, leaving DM intact. A 0.2 mm oversized donor was trephined, stripped of DM and endothelium, and then sutured onto the recipient bed with 16 interrupted 10–0 nylon sutures.

### FSL-PK Group

For the donor cornea, the posterior side cut was set to be penetrating. A 0.2 mm undersized recipient cornea was trephined with similar settings, except that the depth of the posterior side cut was programmed to begin 70 μm anterior to the endothelium at the thinnest point on the optical coherence tomography pachymetry map (Visante, Carl Zeiss Meditec, Inc., Dublin, CA). In the operating room, the adhesions along the cut surface of the recipient cornea were dissected with a Sinskey hook (Rhein Medical, Tampa, FL), and the residual posterior stroma was cut with corneal scissors. The pupil was constricted with miotic (pilocarpine 1 %), and viscoelastic material was put into the anterior chamber to protect the lens. The donor button was then placed on the recipient and sutured with 16 interrupted 10–0 nylon sutures.

Tobramycin-dexamethasone ointment was applied to the eye at the end of the surgery. Eyes were patched and shielded. Postoperatively, patients were commenced on tobramycin- dexamethasone eyedrops with one drop 3 hourly, and slowly tapered over several months. Sutures were removed as clinically indicated.

### Statistical analysis

Data were presented as the Mean ± SE. The data were analyzed by SPSS 17.0 (SPSS, Chicago, IL, USA). Visual acuity was converted to logarithm of the minimum angle of resolution (LogMAR) before statistical analysis. For quantitative variables, independent Student *t* test was used to compare between the FSL-DALK group and the FSL-PK group, and analysis of variance (ANOVA) was used to compare the 3 subgroups. Qualitative variables were analyzed using the *Chi-square* test. Kaplan-Meier functions were constructed to assess the probability of graft survival, and the log rank statistic was used to assess difference between the FSL-DALK and the FSL-PK group. A *P* value less than 0.05 was considered statistically significant.

## Results

**Fig. 1:**
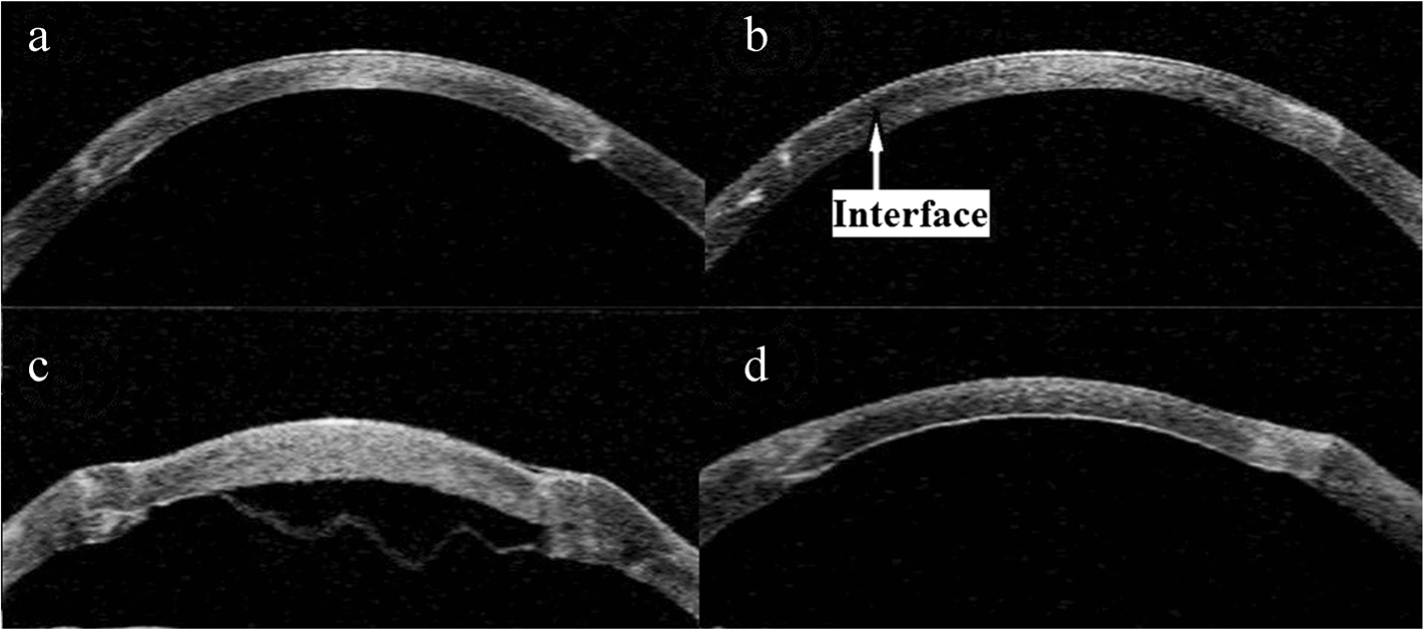
Anterior segment OCT image. **a** Postoperative photograph of one eye underwent femtosecond laser-assisted penetrating keratoplasty (FSL-PK) group at 3 months. **b** Postoperative photograph of one eye underwent femtosecond laser-assisted deep anterior lamellar keratoplasty without baring Descemet’s membrane (FSL-DALKa) at 3 months. **c** Three days after femtosecond laser-assisted deep anterior lamellar keratoplasty baring Descemet’s membrane (FSL-DALKb), one eye developed a Descemet’s membrane detachment and double anterior chamber. **d** 1 month after intracameral air injection with Descemet’s membrane reattached

The corneal profile after surgery was demonstrated using Visante OCT (Fig. 1).

### Patient demographics

Table 1 shows the baseline comparison of the FSL-DALK group with the FSL-PK group. As shown, no statistically significant differences were found between the two groups in terms of gender, age, BCVA, spherical equivalent (SE), or astigmatism (*P* > 0.05).

**Table 1:** Demographic data at baseline

Variables	FSL-DALK	FSL-PK	*P* value
N	28	12	
Gender (M/F)	24/4	9/3	0.410
Age (years)	24.1 ± 6.5	28.0 ± 6.7	0.095
BCVA (LogMAR)	0.68 ± 0.19	0.63 ± 0.19	0.461
SE (D)	−6.8 ± 3.4	−6.5 ± 2.5	0.785
Astigmatism (D)	7.6 ± 3.2	8.1 ± 2.6	0.612

*FSL-DALK*, femtosecond laser-assisted deep anterior lamellar keratoplasty, *FSL-PK* femtosecond laser-assisted penetrating keratoplasty, *BCVA* best-corrected visual acuity, *logMAR* logarithm of the minimum angle of resolution, *SE* spherical equivalent, *D* diopter

### Visual outcomes

After surgery, BCVA improved significantly in all patients (*P* < 0.001). Table 2 summarizes the comparative outcomes during the follow-up. The BCVA in the FSL-PK group and FSL-DALKb subgroup at 3, 6, 9, and 12 months were significantly better than in the FSL-DALKa subgroup (*P* < 0.05); whereas no significant difference was found between the FSL-DALKb subgroup and the FSL-PK group (*P* > 0.05).

**Table 2:** Comparison of BCVA (LogMAR) of FSL-PK and FSL-DALK subgroups

	FSL-PK	FSL-DALKa	FSL-DALKb	*P* value
Preoperative	0.63 ± 0.19	0.65 ± 0.22	0.71 ± 0.18	0.549
3 months	0.25 ± 0.11	0.40 ± 0.10	0.23 ± 0.15	0.003^*^
6 months	0.23 ± 0.10	0.39 ± 0.10	0.22 ± 0.15	0.002^**^
9 months	0.20 ± 0.11	0.39 ± 0.10	0.19 ± 0.16	0.001^***^
12 months	0.19 ± 0.10	0.38 ± 0.11	0.18 ± 0.13	<0.011^****^

*BCVA* best-corrected visual acuity, *logMAR* logarithm of the minimum angle of resolution, *FSL-PK* femtosecond laser-assisted penetrating keratoplasty, *FSL-DALKa* femtosecond laser-assisted deep anterior lamellar keratoplasty without baring Descemet’s membrane, *FSL-DALKb* femtosecond laser-assisted deep anterior lamellar keratoplasty baring Descemet’s membrane with big-bubble technique

^*^FSL-PK vs FSL-DALKa, *P* = 0.008; FSL-PK vs FSL-DALKb, *P* = 0.555; FSL-DALKa vs FSL-DALKb, *P* = 0.001

^**^FSL-PK vs FSL-DALKa, *P* = 0.004; FSL-PK vs FSL-DALKb, *P* = 0.804; FSL-DALKa vs FSL-DALKb, *P* = 0.001

^***^FSL-PK vs FSL-DALKa, *P* = 0.001; FSL-PK vs FSL-DALKb, *P* = 0.863; FSL-DALKa vs FSL-DALKb, *P* < 0.001

^****^FSL-PK vs FSL-DALKa, *P* < 0.001; FSL-PK vs FSL-DALKb, *P* = 0.786; FSL-DALKa vs FSL-DALKb, *P* < 0.001

### Refractive outcomes

No differences in SE and astigmatism were found between the FSL-DALK and the FSL-PK group, nor between the subgroups of FSL-DALK during the follow-up (*P* > 0.05) (Table 3 and 4).

**Table 3:** Comparison of SE (D) of FSL-PK and FSL-DALK subgroups

	FSL-PK	FSL-DALKa	FSL-DALKb	*P* value
Preoperative	−6.45 ± 2.53	−6.36 ± 4.23	−7.05 ± 2.79	0.823
3 months	−3.29 ± 3.07	−2.84 ± 3.04	−1.90 ± 2.02	0.385
6 months	−2.62 ± 2.23	−2.95 ± 3.05	−1.98 ± 1.95	0.558
9 months	−2.59 ± 2.48	−2.89 ± 2.87	−2.10 ± 2.06	0.697
12 months	−2.64 ± 2.30	−2.38 ± 2.84	−1.96 ± 1.76	0.734

*SE* spherical equivalent, *D* diopter, *FSL-PK* femtosecond laser-assisted penetrating keratoplasty, *FSL-DALKa* femtosecond laser-assisted deep anterior lamellar keratoplasty without baring Descemet’s membrane, *FSL-DALKb* femtosecond laser-assisted deep anterior lamellar keratoplasty baring Descemet’s membrane with big-bubble technique

**Table 4:** Comparison of astigmatism (D) of FSL-PK and FSL-DALK subgroups

	FSL-PK	FSL-DALKa	FSL-DALKb	*P* value
Preoperative	8.12 ± 2.62	8.05 ± 3.95	7.23 ± 2.56	0.690
3 months	4.62 ± 1.58	4.90 ± 1.73	4.19 ± 1.57	0.511
6 months	4.21 ± 0.97	4.87 ± 1.78	3.69 ± 1.18	0.083
9 months	3.73 ± 0.92	4.72 ± 2.25	3.39 ± 1.02	0.068
12 months	3.97 ± 1.06	4.27 ± 2.43	3.51 ± 0.95	0.445

*D* diopter, *FSL-PK* femtosecond laser-assisted penetrating keratoplasty, *SL-DALKa* femtosecond laser-assisted deep anterior lamellar keratoplasty without baring Descemet’s membrane, *FSL-DALKb* femtosecond laser-assisted deep anterior lamellar keratoplasty baring Descemet’s membrane with big-bubble technique

### Endothelial cell density

**Fig. 2:**
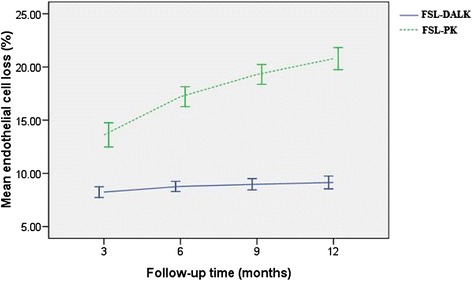
Endothelial cell loss after surgery. Mean endothelial cell loss (%) after femtosecond laser-assisted deep anterior lamellar keratoplasty (FSL-DALK) versus femtosecond laser-assisted penetrating keratoplasty (FSL-PK)

Before surgery, ECD was measured in all donor corneas in the FSL-PK group. The mean preoperative ECD was 2569 ± 329 cells/mm^2^ and 2403 ± 155 cells/mm^2^ in the FSL-DALK and FSL-PK group, respectively (*P* = 0.137). In both groups, a progressive and statistically significant reduction in ECD was found during the follow-up (*P* < 0.05). In the FSL-DALK group, the mean postoperative endothelial cell loss was 8.19 %, 8.71 %, 8.98 %, and 9.12 % at 3 months, 6 months, 9 months, and 12 months, respectively. In the FSL-PK group, the mean postoperative endothelial cell loss was 13.62 %, 17.21 %, 19.30 %, and 20.79 % at 3 months, 6 months, 9 months, and 12 months, respectively. Significant higher endothelial cell loss was observed in the FSL-PK group (*P* < 0.001; Fig. 2). Comparing the subgroups, the mean endothelial cell losses were not different between the FSL-DALKa and FSL-DALKb groups during the follow-up (*P* > 0.05).

### Complications

**Fig. 3:**
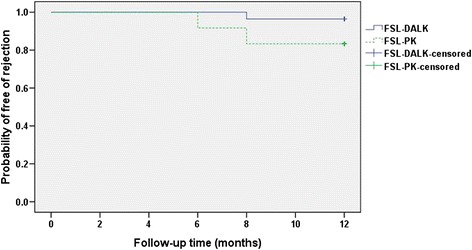
Survival rates after surgery. Kaplan-Meier rejection-free survival curves of eyes underwent femtosecond laser-assisted deep anterior lamellar keratoplasty (FSL-DALK) versus femtosecond laser-assisted penetrating keratoplasty (FSL-PK). Although the FSL-DALK group seems to have fewer rejections, the difference between the curves did not reach statistical significance (*P* = 0.144, log rank test)

DM microperforation occurred in 2 eyes in the FSL-DALKb group which did not require conversion to FSL-PK. No intraoperative complication occurred in the FSL-PK. Graft rejection occurred in 2 eyes in the FSL-PK group, and one episode of stromal rejection occurred in the FSL-DALKa group which was resolved with topical corticosteroid (Fig. 3, *P* = 0.144, log rank test). Of the 2 eyes in the FSL-PK group, one resolved successfully with topical corticosteroid, whereas regrafting was necessary in the other. Post-operative new-onset secondary glaucoma was diagnosed in 3 eyes in the FSL-PK group, and it was successfully controlled with topical medications only. One eye developed a DM detachment and double anterior chamber in the FSL-DALKb group 3 days after surgery, which was managed by intracameral air injection and achieved complete tamponade of the DM within 1 week (Fig. 1c, d). After 1 month, the eye obtained a BCVA (LogMAR) of 0.10, and DM remained attached during the follow-up. Other postoperative complications included epitheliopathy (2 eyes in the FSL-PK group versus 1 eye in the FSL-DALKa group) and graft resuturing due to wound dehiscence (1 eye in the FSL-DALKa group). Suture removal was performed as clinically indicated: at 6 months in the FSL-DALK group and at 12 months in the FSL-PK group. Loose sutures were removed upon diagnosis in all eyes.

## Discussion

After surgery, BCVA improved significantly in all eyes, and we also found that FSL-DALK baring DM with the big-bubble technique provided matching visual acuity in comparison with PK, whereas FSL-DALK without baring DM, which left behind a layer of the recipient bed, produced poorer visual outcomes. This finding was comparable to previous reports, although the cut of cornea in these reports was in use of mechanical trephine instead of FSL [11, 12]. Ardjomand et al. demonstrated that the quality of vision after keratoplasty was correlated to the thickness of the residual recipient stromal bed [11]. They found that eyes with a recipient stromal bed thickness of less than 20 μm had comparable visual acuity with eyes that had undergone PK, whereas those with a recipient thickness of more than 80 μm had significantly reduced visual acuity. This finding was also comparable to the study by Han et al. [12], in which they found that DALK with modified Anwar technique provided comparable visual acuity in comparison with PK, whereas those with manual DALK had poorer visual outcomes.

The major long-term advantage of DALK surgery over PK relates to long-term preservation of host corneal endothelial cell. A report by Reinhart et al. showed that continued or accelerated endothelial cell loss after DALK surgery did not seem to occur after 6 months as it does after PK, and that endothelial cell loss after the immediate postoperative time period was likely to mimic the gradual endothelial cell decrease of a normal cornea [13]. In this study, we found a progressive reduction in ECD in both groups after the surgery, whereas a significant higher endothelial cell loss was observed in the FSL-PK group. At the last follow-up, the mean endothelial cell loss was 20.79 % in the FSL-PK group which was much higher than 9.12 % in the FSL-DALK group. In a similar study, Sogutlu et al. prospectively compared DALK and PK over a 2-year period and demonstrated a slower endothelial cell loss in the DALK group [14]. Some studies have shown that PK leads to a precipitated rate of endothelial cell loss which may contribute to graft failure in a portion of PK procedures [15]. In this study, the Kaplan-Meier curve showed no significant difference in graft survival between the FSL-DALK and the FSL-PK groups. A report from the American Academy of Ophthalmology showed that there was no significant difference in graft survival between DALK and PK, whereas a recent prospective cohort study by Coster showed a worse survival of DALK than PK [13, 16]. Nevertheless, the risk of graft failure should be kept in mind when evaluating a patient with keratoconus.

Refractive outcomes improved significantly in all eyes after surgery. The greatest changes in SE and astigmatism were at 3 months. At the last follow-up, mean SE and astigmatism in the FSL-PK group were −2.64 D and 3.97 D, respectively. Mean SE and astigmatism in the FSL- DALK group were −2.14 D and 3.84 D at the last follow-up, respectively. The refractive outcomes in the FSL-DALK group were comparable to the results reported by Buzzonetti et al. [8] but inferior to the results reported by Shehadeh-Mashor et al. [17]. In our study, no differences in SE and astigmatism were found between the FSL-DALK and the FSL-PK group and between subgroups during the follow-up. Many studies showed similar postoperative refractive outcomes between DALK and PK [12, 18]. A report from the American Academy of Ophthalmology, as well, demonstrated that there was no advantage to DALK for refractive error outcomes compared with PK [13]. In contrast, a study by Funnell found that corneal astigmatism was significantly less in the DALK than in the PK [19]. In our study, we use the FSL trephination 90° to the surface, which results in a more physiological donor-recipient matching and requires less tight sutures than manual trephination. This may account for the comparable astigmatism between the FSL-PK and the FSL-DALK in this study.

In our study, no intraoperative complication occurred in the FSL-PK group. DM microperforation occurred in 2 eyes (7.1 %) in the FSL-DALKb group, which was lower than manual trephination reported by other studies [20, 21]. A consideration of the increased risk of complication (perforation), even with FSL, should be weighed when considering the FSL-DALKb procedure. Also, learning curve is another consideration. DM perforation is the main intraoperative complication in DALKb. The use of the FSL in DALK avoids manual trephination and allows maintenance of predefined corneal depth very close to endothelium and insertion of the air needle by following the plane between the lamellar and posterior laser side cuts. Injection of air at this precisely predefined pre-DM plane may facilitate the big bubble formation with full baring of DM [8, 22]. Even with FSL and big-bubble technique, DM microperforation may happen. Because microperforation may be created by a very tiny forceps tip when remove the lamellar from the DM. However, one would expect a lower DM perforation with this technique. Although the two cases did not convert to FSL-PK, one case developed DM detachment and pseudo double anterior chamber 3 days after surgery (Fig. 1c). Double anterior chamber is usually a consequence of DM perforation, and can be managed by intracameral air or synthetic gas injection [13, 23]. This case was managed by intracameral air injection with successful reattachment of DM within 1 week. After 1 month, the eye obtained a BCVA (LogMAR) of 0.10, and DM remained attached during the follow-up (Fig. 1d).

DALK is reported to have fewer postoperative complications than PK [19], however, our study did not do the statistic because of the sparse data. Graft rejection occurred in 2 eyes in the FSL-PK group (2/12), and one episode of stromal rejection occurred in the FSL-DALK group (1/28) which was resolved with topical corticosteroid. Of the 2 eyes in the FSL-PK group, one resolved successfully with topical corticosteroid, whereas regrafting was necessary in the other. Postoperative new-onset secondary glaucoma was diagnosed in 3 eyes in the FSL-PK group compared with no eye in the FSL-DALK group, and it was successfully controlled with topical medications only. Topical steroid treatment in the FSL-DALK group was shorter than in the FSL-PK group, and the reduced duration of steroid treatment would reduce the likelihood of steroid induced ocular hypertension. Other postoperative complications included epitheliopathy (2 eyes in the FSL-PK group versus 1 eye in the FSL-DALK group) and graft resuturing due to wound dehiscence (1 eye in the FSL-DALK group).

The limitations of this study include its retrospective nature, small sample size, absence of randomization, and short follow-up period. From the results of the present and other studies, it seems that FSL-DALK with the big-bubble technique should be considered as the best technique for keratoconus whose endothelium is not compromised [7, 8, 20, 22, 24]. However, further large randomized prospective study is needed to validate the benefits of FSL-DALK baring DM with big-bubble technique.

## Conclusions

In summary, the outcomes of this case series supported the benefits and safety of FSL-DALK baring DM with big-bubble technique, which provided comparable visual outcomes and less endothelial cell loss as compared with FSL-PK. FSL-DALK baring DM with big-bubble technique is a viable option for keratoconus whose endothelium is not compromised. However, FSL-PK should be indicated for keratoconus with previous hydrops and scars. For more convincing, larger and prospective studies are required.

## Abbreviations

FSL-DALK: Femtosecond laser-assisted deep anterior lamellar keratoplasty
FSL-PK: Femtosecond laser-assisted penetrating keratoplasty
BCVA: Best-corrected visual acuity
DM: Descemet’s membrane
LogMAR: Logarithm of the minimum angle of resolution
OCT: Ocular coherence tomography
SE: Spherical equivalent
ECD: Endothelial cell density

## References

[1] Troutman RC, Lawless MA (1987). Penetrating keratoplasty for keratoconus. Cornea.

[2] Pramanik S, Musch DC, Sutphin JE, Farjo AA (2006). Extended long-term outcomes of penetrating keratoplasty for keratoconus. Ophthalmology.

[3] Thompson RW, Price MO, Bowers PJ, Price FW (2003). Long-term graft survival after penetrating keratoplasty. Ophthalmology.

[4] Shimmura S, Tsubota K (2006). Deep anterior lamellar keratoplasty. Curr Opin Ophthalmol.

[5] Alio JL, Shah S, Barraquer C, Bilgihan K, Anwar M, Melles GR (2002). New techniques in lamellar keratoplasty. Curr Opin Ophthalmol.

[6] Tan DT, Ang LP (2006). Modified automated lamellar therapeutic keratoplasty for keratoconus: a new technique. Cornea.

[7] Anwar M, Teichmann KD (2002). Big-bubble technique to bare Descemet’s membrane in anterior lamellar keratoplasty. J Cataract Refract Surg.

[8] Buzzonetti L, Petrocelli G, Valente P (2012). Femtosecond laser and big-bubble deep anterior lamellar keratoplasty: a new chance. J Ophthalmol.

[9] Chamberlain WD, Rush SW, Mathers WD, Cabezas M, Fraunfelder FW (2011). Comparison of femtosecond laser-assisted keratoplasty versus conventional penetrating keratoplasty. Ophthalmology.

[10] Yoo SH, Kymionis GD, Koreishi A, Ide T, Goldman D, Karp CL, O’Brien TP, Culbertson WW, Alfonso EC (2008). Femtosecond laser-assisted sutureless anterior lamellar keratoplasty. Ophthalmology.

[11] Ardjomand N, Hau S, McAlister JC, Bunce C, Galaretta D, Tuft SJ, Larkin DF (2007). Quality of vision and graft thickness in deep anterior lamellar and penetrating corneal allografts. Am J Ophthalmol.

[12] Han DC, Mehta JS, Por YM, Htoon HM, Tan DT (2009). Comparison of outcomes of lamellar keratoplasty and penetrating keratoplasty in keratoconus. Am J Ophthalmol.

[13] Reinhart WJ, Musch DC, Jacobs DS, Lee WB, Kaufman SC, Shtein RM (2011). Deep anterior lamellar keratoplasty as an alternative to penetrating keratoplasty a report by the american academy of ophthalmology. Ophthalmology.

[14] Sogutlu Sari E, Kubaloglu A, Unal M, Pinero D, Bulut N, Erol MK, Özertürk Y (2013). Deep anterior lamellar keratoplasty versus penetrating keratoplasty for macular corneal dystrophy: a randomized trial. Am J Ophthalmol.

[15] Bohringer D, Reinhard T, Spelsberg H, Sundmacher R (2002). Influencing factors on chronic endothelial cell loss characterised in a homogeneous group of patients. Br J Ophthalmol.

[16] Coster DJ, Lowe MT, Keane MC, Williams KA (2014). A Comparison of Lamellar and Penetrating Keratoplasty Outcomes: A Registry Study. Ophthalmology.

[17] Shehadeh-Mashor R, Chan CC, Bahar I, Lichtinger A, Yeung SN, Rootman DS (2013). Comparison between femtosecond laser mushroom configuration and manual trephine straight-edge configuration deep anterior lamellar keratoplasty. Br J Ophthalmol.

[18] Bahar I, Kaiserman I, Srinivasan S, Ya-Ping J, Slomovic AR, Rootman DS (2008). Comparison of three different techniques of corneal transplantation for keratoconus. Am J Ophthalmol.

[19] Funnell CL, Ball J, Noble BA (2006). Comparative cohort study of the outcomes of deep lamellar keratoplasty and penetrating keratoplasty for keratoconus. Eye (Lond).

[20] Borderie VM, Sandali O, Bullet J, Gaujoux T, Touzeau O, Laroche L (2012). Long-term results of deep anterior lamellar versus penetrating keratoplasty. Ophthalmology.

[21] Kubaloglu A, Sari ES, Unal M, Koytak A, Kurnaz E, Cinar Y, Ozertürk Y (2011). Long-term results of deep anterior lamellar keratoplasty for the treatment of keratoconus. Am J Ophthalmol.

[22] Huang T, Zhang X, Wang Y, Zhang H, Huand A, Gao N (2012). Outcomes of deep anterior lamellar keratoplasty using the big-bubble technique in various corneal diseases. Am J Ophthalmol.

[23] Mannan R, Jhanji V, Sharma N, Titiyal JS, Vajpayee RB (2007). Intracameral C(3)F(8) injection for descemet membrane detachment after phacoemulsification in deep anterior lamellar keratoplasty. Cornea.

[24] Buzzonetti L, Laborante A, Petrocelli G (2011). Refractive outcome of keratoconus treated by combined femtosecond laser and big-bubble deep anterior lamellar keratoplasty. J Refract Surg.

